# The efficacy of three-ball breathing apparatus exercise based on the concept of pulmonary rehabilitation in patients after lung cancer surgery

**DOI:** 10.1186/s13019-023-02307-0

**Published:** 2023-07-06

**Authors:** Qiang Xu, Zi-Qing Shen, Kun-Peng Feng, Chun Xu, Cheng Ding, Chang Li, Sheng Ju, Jun Chen, Shu Pan, Jun Zhao

**Affiliations:** 1 Department of Thoracic Surgery, The First Affiliated Hospital of Soochow University, Medical College of Soochow University, Suzhou, 215000 China; 2 Institute of Thoracic Surgery, The First Affiliated Hospital of Soochow University, Suzhou, China

## Abstract

**Background:**

Postoperative patients with lung cancer mostly experience different degrees of dyspnea and decreased activity tolerance, and these symptoms all significantly affect postoperative quality of life. The concept of pulmonary rehabilitation applicable to patients with chronic respiratory diseases is also applicable to patients with postoperative lung cancer. The current application of postoperative pulmonary rehabilitation for lung cancer is inconsistent, and reliable guidelines are lacking. The purpose of this study was to further verify the efficacy and feasibility of postoperative pulmonary rehabilitation for lung cancer patients, and to find a suitable local pulmonary rehabilitation program for postoperative patients with lung cancer that is clinically promoted in our department through this study.

**Methods:**

We collected the clinical data of patients undergoing video-assisted thoracoscopic surgery (VATS) wedge resection or lobectomy. The patients were divided into rehabilitation group (using three-ball breathing apparatus after discharge) and control group (routine follow-up after discharge) according to whether the patients were trained with three-ball breathing apparatus after operation. The detailed method using three-ball apparatus is as follows. To begin with, patients are required to put themselves in a comfortable position. Then, after the three-ball breathing apparatus put on the same plane of their eyes, patients hold the tube in their mouth closely and control their breath slowly. When patients inhale to their largest extent, the balls will rise up accordingly. Then they exhale. The evaluation results of pulmonary function, activity tolerance, anxiety scores and others were collected. All data was gathered at the First Affiliated Hospital of Soochow University. The effects of pulmonary rehabilitation training on wedge resection and lobectomy were compared.

**Results:**

A total of 210 patients were included in this study, including 126 patients with VATS wedge resection and 84 patients with VATS lobectomies. No discrepancy was noticed when FEV_1_ loss between two groups were compared in the wedge resection patients, and the same results were also shown in patients undergoing lobectomy (12.8% ± 2.0% vs. 12.7% ± 1.9%, *P* = 0.84, wedge resection; 12.6% ± 2.9% vs. 12.1% ± 1.8%, *P* = 0.37, lobectomy). The loss of FVC in the control group was greater than that in the rehabilitation group for patients undergoing lobectomy (11.7% ± 5.2%, vs. 17.1% ± 5.6%, *P* < 0.001, lobectomy). No difference was found in the wedge resection patients between the control and rehabilitation groups (6.6% ± 2.8%, vs. 6.4% ± 3.2%, *P* = 0.76, lobectomy). Moreover, all patients showed no significant difference in 6MWD regardless of surgical procedure and with or without breathing exercises at T3 (392.6 ± 50.6 m, rehabilitation group vs. 394.0 ± 46.6 m, control group. *P* = 0.87, wedge resection; 381.3 ± 38.9 m, rehabilitation group vs. 369.1 ± 49.3 m, control group. *P* = 0.21, lobectomy).

**Conclusions:**

For patients after thoracoscopic pulmonary wedge resection, the use of three-ball apparatus did not significantly improve postoperative pulmonary function and activity tolerance, dyspnea, and anxiety symptoms. In patients after thoracoscopic lobectomy, respiratory trainers were able to improve postoperative lung function but were unable to significantly improve dyspnea and anxiety symptoms. There was a significant benefit for the use of three-ball apparatus in patients after thoracoscopic lobectomy, whereas there was no significant benefit for the use of respiratory trainers after wedge resection.

*Registry*: Medical Ethics Committee of the First Affiliated Hospital of Soochow University. Registration number: no. 2022455.

## Introduction

Lung cancer is one of the most common several malignant tumors worldwide [1], and surgical treatment remains the mainstay of lung cancer treatment [2]. Postoperative patients with lung cancer mostly experience dyspnea of varying degrees, or shortness of breath and decreased activity tolerance, and some patients then experience depression and anxiety and other manifestations [3, 4], these symptoms all significantly affect the postoperative health-related quality of life (HRQoL) [5, 6]. Even partial lung cancer patients with COPD will have more symptoms such as respiratory suffering, low exercise capacity and depression. And evidence suggests that postoperative dyspnea and poor exercise tolerance are associated with reduced survival in lung cancer patients [7, 8].

As the concept of enhanced recovery after surgery (ERAS) is gradually accepted by more surgeons. Thoracic surgeons will focus more attention on the intraoperative as well as the postoperative hospitalization period in the rehabilitation of surgical patients [9], such as the smaller surgical trauma in the operation, the encouragement of postoperative patients to cough and discharge more phlegm, and so on, the aim is to achieve a faster and better recovery of patients, and eventually a better cure for their discharge [10–12]. However, some thoracic surgeons do not focus on the period after discharge of patients undergoing lung cancer surgery and before hospitalization. it has been well documented that prehospital exercise training has the potential to adjust nonsurgical candidates to surgical candidates [13, 14]; several investigators have also found that patients after lung cancer surgery are discharged from the hospital with resistance training, endurance training, and other modalities, which can increase their walking endurance and can also reduce the dyspnea of patients [15–17]. Part of the investigators' findings also allowed a new idea to emerge for thoracic surgeons, which was pulmonary rehabilitation.

In previous cognition, pulmonary exercise training was only suitable for those with chronic respiratory diseases, such as COPD and so on [18]. However, with the publication of the latest guidelines on pulmonary rehabilitation and the discovery of some clinical studies, the concept of pulmonary rehabilitation applicable to patients with chronic respiratory diseases is also applicable to patients with lung cancer after surgery [8]. And American Thoracic Society (ATS) and European Respiratory Society (ERS) in their statement mentions that exercise training based on the concept of pulmonary rehabilitation is not light adapted for patients with COPD but also other respiratory diseases, such as lung cancer, cystic fibrosis, bronchiectasis, neuromuscular disease, etc. The specific definitions of pulmonary rehabilitation in the guidelines are as follows:” *Pulmonary rehabilitation is a comprehensive intervention based on a thorough patient assessment followed by patient-tailored therapies, which include, but are not limited to, exercise training, education, and behavior change, designed to improve the physical and psychological condition of people with chronic respiratory disease and to promote the long-term adherence of health-enhancing behaviors.*”

The main form of pulmonary rehabilitation is exercise training [19], drugs and other modalities can make the effect of exercise training better [20, 21]. Exercise training consisted of endurance training, interval training, resistance/strength training, inspiratory muscle training. Evidence demonstrating that preoperative pulmonary rehabilitation can optimize exercise tolerance and overall medical stability in individuals prior to lung cancer resection [13, 14, 22, 23], preoperative short-term pulmonary rehabilitation is confirmed to be feasible and effective. But the current application of pulmonary rehabilitation after lung cancer is inconsistent [24–28], the reason may be that the current application program of pulmonary rehabilitation is mostly borrowed in view of its application program in COPD patients, lack of reliable guidelines. However, most of the current studies on postoperative pulmonary rehabilitation are small sample size, which leads to heterogeneity and unreliability of the results.

The purpose of this study was to further verify the efficacy and feasibility of postoperative pulmonary rehabilitation for lung cancer patients, and through this study to find a suitable local region pulmonary rehabilitation program for postoperative patients with lung cancer in our department for clinical promotion.

## Method

This retrospective study was approved by the ethics committee of the First Affiliated Hospital of Soochow University.

### Patients

**Fig. 1:**
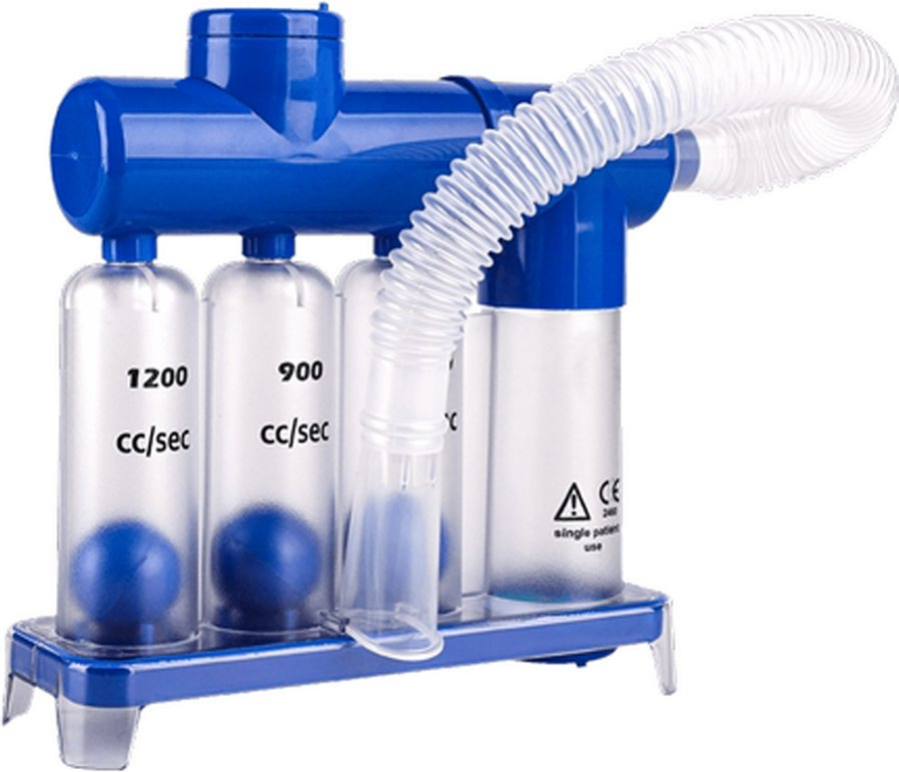
Three-ball breathing apparatus

**Fig. 2:**
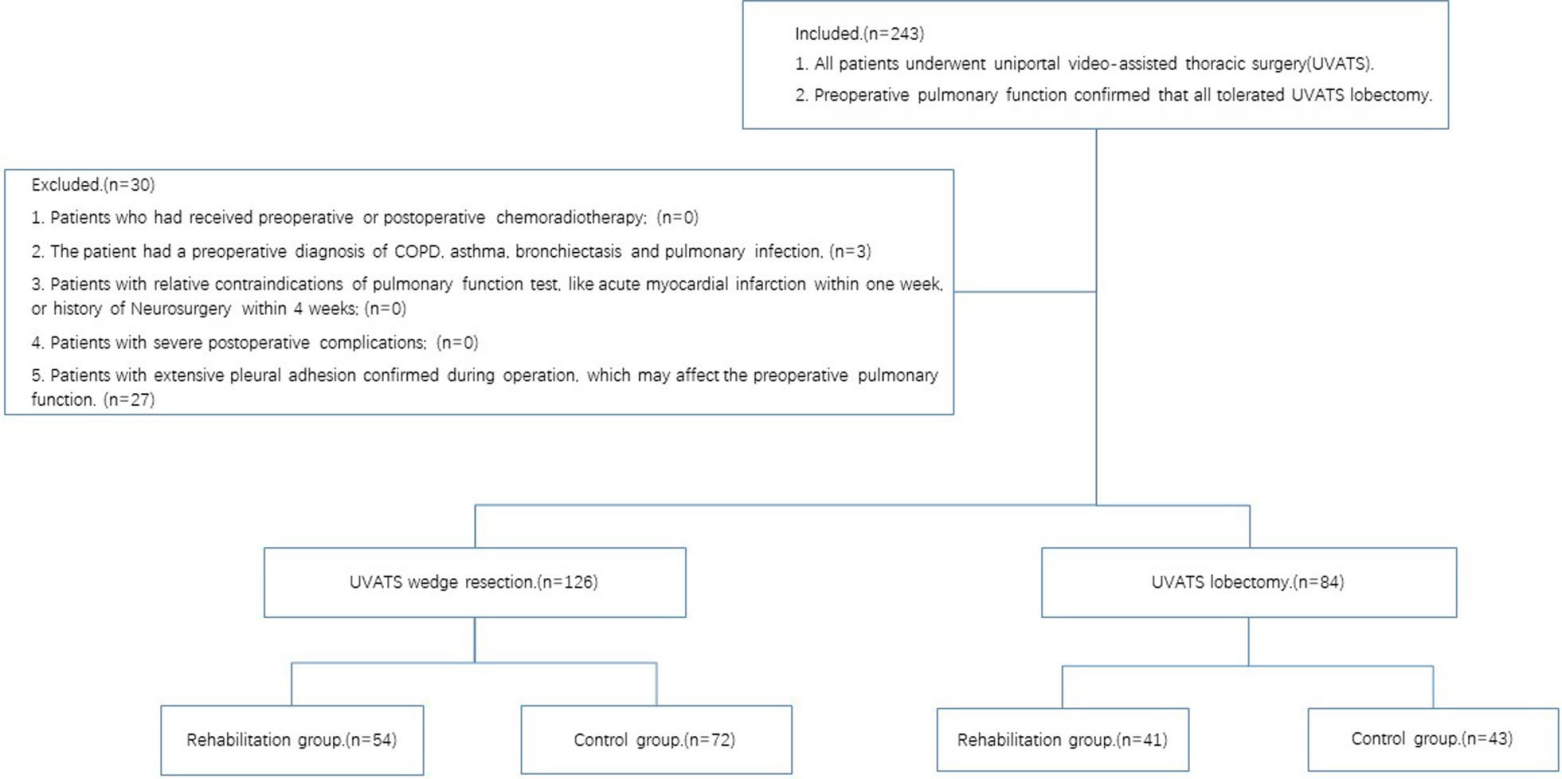
Flow Chart. (Rehabilitation Group: using three-ball breathing apparatus after discharge; Control Group: routine follow up after discharge)

Exercise therapy is the core content and cornerstone of pulmonary rehabilitation, which mainly includes endurance training, strength training, and respiratory muscle rehearsal [8]. Due to the lack of guidelines for the standards of endurance training and strength training, and follow-up is difficult. Some patients with lung cancer in our department used three-ball breathing apparatus (Fig. 1) for respiratory function exercise after discharge. The detailed method using three-ball apparatus is as follows. To begin with, patients are required to put themselves in a comfortable position. Then, after the three-ball breathing apparatus put on the same plane of their eyes, patients hold the tube in their mouth closely and control their breath slowly. When patients inhale to their largest extent, the balls will rise up accordingly. Then they exhale. Therefore, the patients included in this study include the rehabilitation group (using three-ball breathing apparatus after discharge) and the control group (routine follow up after discharge). We retrospectively collected patients who underwent thoracoscopic lung resection surgery at the Department of thoracic surgery, the First Affiliated Hospital of Soochow University between February and April 2022 (Fig. 1). A total of 210 patients were included in this study (Fig. 2), including 126 for VATS wedge resection and 84 for VATS lobectomy. Patients with both surgical approaches were divided into rehabilitation (using three-ball breathing apparatus after discharge) and control groups (routine follow up after discharge) according to whether they performed three-ball breathing apparatus exercise after discharge.

### Outcome measures

All assessments were performed at baseline and weeks 4 (T1), 12 (T2), and 24 (T3). Lung function data (FVC, FEV1) were collected by spirometry, and loss of lung function was subsequently calculated. Pulmonary function tests include: FVC, FEV1. The pulmonary function loss was calculated as follows (take FVC loss as an example): FVC loss = (preoperative FVC—postoperative FVC)/preoperative FVC × 100% [29]. Perceived severity of breathlessness was measured on a 0–10 modified BORG Scale was used for assessment of quality of life in patients with chronic airflow limitations. Hospital Anxiety and Depression Scale (HADS) served as measuring anxiety and depression [30, 31]. All questionnaire scores were obtained by outpatient questioning at the time of patient review.

### Data analysis

We used SPSS 25.0 software (Statistical Package for the Social Sciences, Chicago, IL, USA) for data analysis. All data results were expressed as mean value ± standard deviation. Independent sample *t*-test was applied for the measurement data conforming to normal distribution while Mann–Whitney U test was used for that not conforming to normal distribution. When *P* value of less than 0.05, statistical significance was accepted.

## Results

### Sociodemographic and clinical characteristics

A total of 210 patients were included in this study. A statistical study was performed by subgroup according to the type of surgery performed (126 wedge resections and 84 lobectomies). The wedge resection group was divided into rehabilitation (54 patients, using three-ball breathing apparatus after discharge) and control groups (72 patients, routine follow up after discharge) based on the presence or absence of respirator training, and the lobectomy group performed the same procedure (rehabilitation group: 41patients; control group: 43 patients). Demographic and other clinical data are shown together in Table 1.

**Table 1:** Clinical data of all patients

	UVATS wedge resection		*P* value	UVATS lobectomy		*P* value
	Rehabilitation group (n = 54)	Control group (n = 72)		Rehabilitation group (n = 41)	Control group (n = 43)	
Age(year)	42. ± 12.2	42.9 ± 13.3	0.175	40.5 ± 15.5	43.0 ± 10.8	0.382
Gender						
Female	40	51		23	28	
Male	14	21	0.688	18	15	0.716
Smoking history						
Yes	2	6		3	3	
No	52	66	0.292	38	40	0.952
Pulmonary function (L/min)						
FEV_1_						
Baseline	2.20 ± 0.45	2.18 ± 0.42	0.82	2.23 ± 0.35	2.22 ± 0.37	0.96
T3	1.91 ± 0.40	1.90 ± 0.37	0.84	1.95 ± 0.32	1.95 ± 0.33	0.92
FEV_1_ loss (%)	12.8 ± 2.0	12.7 ± 1.9	0.84	12.6 ± 2.9	12.1 ± 1.8	0.37
FVC						
Baseline	2.63 ± 0.55	2.59 ± 0.49	0.70	2.71 ± 0.40	2.56 ± 0.44	0.11
T3	2.45 ± 0.51	2.42 ± 0.46	0.75	2.39 ± 0.41	2.12 ± 0.38	< 0.01
FVC loss (%)	6.6 ± 2.8	6.4 ± 3.2	0.76	11.7 ± 5.2	17.1 ± 5.6	< 0.001
6MWD (m)						
Baseline	385.0 ± 54.9	394.2 ± 56.0	0.35	397.4 ± 50.1	396.8 ± 63.2	0.95
T1	364.4 ± 43.7	375.2 ± 53.9	0.23	336.7 ± 47.8	325.5 ± 50.1	0.29
T2	384.1 ± 45.6	384.9 ± 38.8	0.91	367.3 ± 41.0	335.4 ± 53.9	< 0.01
T3	392.6 ± 50.6	394.0 ± 46.6	0.87	381.3 ± 38.9	369.1 ± 49.3	0.21
Modified BORG Scale						
Baseline	0	0		0	0	
T1	3.37 ± 1.12	3.47 ± 1.17	0.62	4.14 ± 1.10	4.58 ± 1.00	0.63
T2	1.27 ± 1.20	1.59 ± 1.18	0.13	3.73 ± 0.74	3.67 ± 0.80	0.73
T3	1.11 ± 0.92	1.16 ± 0.97	0.74	2.26 ± 1.65	1.00 ± 0.89	< 0.001
HADS						
Baseline	0	0		0	0	
T1	10.50 ± 3.07	9.76 ± 3.10	0.18	9.73 ± 3.32	9.95 ± 3.36	0.76
T2	4.77 ± 1.67	4.45 ± 1.70	0.29	4.70 ± 1.60	4.37 ± 1.67	0.35
T3	2.79 ± 1.90	3.22 ± 2.00	0.23	2.58 ± 1.80	2.93 ± 2.16	0.43

Preoperative pulmonary function and other observation indicators are shown in Table 1. Pulmonary function tests were performed preoperatively and at the sixth month after surgery. No discrepancy was noticed when FEV_1_ loss between two groups were compared in the wedge resection patients, and the same results were also shown in patients undergoing lobectomy (12.8% ± 2.0% vs. 12.7% ± 1.9%, *P* = 0.84, wedge resection; 12.6% ± 2.9% vs. 12.1% ± 1.8%, *P* = 0.37, lobectomy). The loss of FVC in the control group was greater than that in the rehabilitation group for patients undergoing lobectomy (11.7% ± 5.2%, vs. 17.1% ± 5.6%, *P* < 0.001, lobectomy). Interestingly in the wedge resection patients, no such difference was found between the control and rehabilitation groups (6.6% ± 2.8%, vs. 6.4% ± 3.2%, *P* = 0.76, lobectomy).

All patients showed various degrees of decrease in their 6-min walk distance (6MWD) after surgery. Differences between the rehabilitation and control groups were only found in patients who had undergone lobectomy at T2 (367.3 ± 41.0 m, rehabilitation group vs. 335.4 ± 53.9 m, control group. *P* < 0.01). Moreover, all patients showed no significant difference in 6MWD regardless of surgical procedure and with or without breathing exercises at T3 (392.6 ± 50.6 m, rehabilitation group vs. 394.0 ± 46.6 m, control group. *P* = 0.87, wedge resection; 381.3 ± 38.9 m, rehabilitation group vs. 369.1 ± 49.3 m, control group. *P* = 0.21, lobectomy). Dyspnea and anxiety scores showed a similar pattern in all enrolled patients, with the highest symptom scores at T1 in all patients, followed by a stepwise decline at T2 to T3. However, all patients still had higher symptom scores at T3 than at baseline. Among all enrolled patients at T3, the rehabilitation group had higher Borg scores than the control group among the patients after wedge resection (2.26 ± 1.65, rehabilitation group vs. 1.00 ± 0.89, control group. *P* < 0.001), no significant difference in anxiety scores, and no significant difference between the rehabilitation group and the control group in comparing symptom scores among the patients after lobectomy.

## Discussion

Prolonging survival is not the only purpose of lung cancer surgery, and improving the quality of patients' survival after surgery is also important [32]. However, surgery may cause some effects on human body, such as postoperative pain, pleural reaction, dyspnea, and reduction of lung volume [33]. In response to these situations, the body spontaneously reinforces the function of accessory respiratory muscles, forming abnormal breathing. However, the presence of abnormal respiration may aggravate respiratory muscle fatigue and finally lead to carbon dioxide accumulation, severe dyspnea, etc. [34]. Different degrees of dyspnea can affect the quality of life of patients and lead to different degrees of depression or anxiety [35].

The results of this study show insignificant improvement in lung function with long-term application of respiratory trainers in patients after wedge resection of the lung. The use of breathing trainers does not reduce the loss of FVC in patients, which may be related to the less lung tissue resected in the wedge resection procedure and the low amount of stapler used, ultimately resulting in less effect on lung volume. And articles have reported that FVC in patients after wedge resection of the lung can return to the preoperative level at one year postoperatively without any rehabilitation measures after discharge [36]. The loss of FEV_1_ in the study also showed the same result. The FEV_1_ index of patients with respiratory training was not significantly improved compared with patients without respiratory training. This may be related to the way we perform surgery, because all patients perform UVATS, which has little damage to respiratory muscles, while FEV_1_ is related to the functional integrity of respiratory muscles [37, 38]. The symptom scores of dyspnea and anxiety in both groups were high and not statistically different at the first postoperative reexamination, which may have a great association with pain in the postoperative wound. However, at the third review postoperatively, the symptoms of dyspnea and anxiety had already significantly relieved in both groups, but there was still no statistical difference. So, we think that patients after thoracoscopic wedge resection, respiratory training using a three-ball breathing apparatus may not be able to harvest the improvement of lung function as well as dyspnea, quality of life; or that patients after wedge resection may not need rehabilitation for a long period of time because the effect of wedge resection on patients, whether pulmonary function or quality of life, etc., has not been significant over time.

**Fig. 3:**
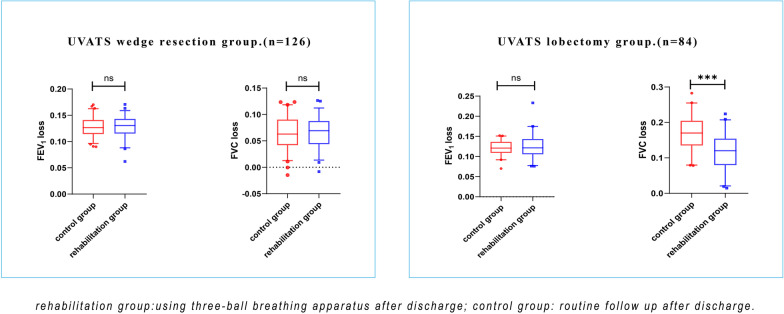
Among lung cancer patients who underwent wedge resection, the amount of lung function loss was not significantly different in the rehabilitation group than in the control group; However, in patients who underwent lobectomy, FVC loss in the rehabilitation group was less than that in the control group, and there was no significant difference in FEV_1_ loss

**Fig. 4:**
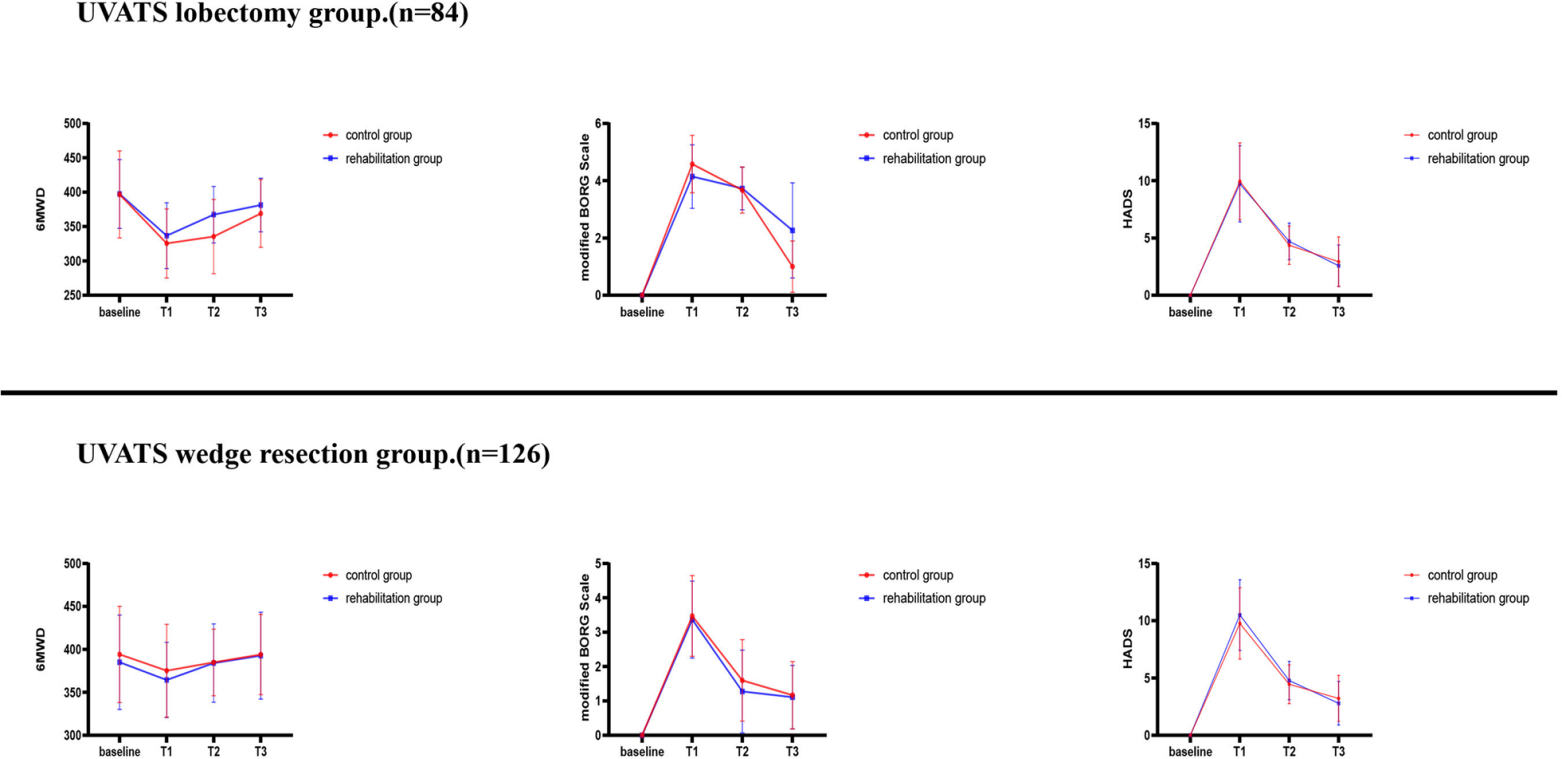
All assessments were performed at baseline and weeks 4 (T1), 12 (T2), and 24 (T3). Rehabilitation group: using three-ball breathing apparatus after discharge; Control group: routine follow up after discharge

The pulmonary function of patients after lobectomy is mainly reflected in the loss of FVC. The loss of FVC of patients with long-term respiratory training is less than that of the other group, while the loss of FEV_1_ has no significant difference between the two groups (Fig. 3). We believe that respiratory training can improve the FVC index of patients after lobectomy (Fig. 4). However, since our study only investigated and followed up patients 24 weeks after operation, it is still unclear whether long-term use of respiratory training can better improve the pulmonary function of patients after lobectomy. No difference was found between the two groups in anxiety scores of patients after lobectomy, but there was a significant difference in BORG scores at T3, the patients in the respiratory trainer group had higher BORG scores. We consider this may be related to patients’ inappropriate methods of using respiratory trainers or the subjectivity of scoring questionnaires. Because patients using respiratory trainers had better lung function than nonusers in terms of lung function outcomes.

## Conclusion

For patients after thoracoscopic pulmonary wedge resection, the use of three-ball breathing apparatus did not significantly improve postoperative pulmonary function and symptoms of activity tolerance, dyspnea, and anxiety. However, in patients after thoracoscopic lobectomy, breathing trainers were able to improve postoperative lung function but were unable to significantly improve dyspnea and anxiety symptoms. There was a significant benefit in the use of three-ball breathing apparatus in patients after thoracoscopic lobectomy, while the patients after wedge resection who use the respiratory trainer have no significant benefits. The application of pulmonary rehabilitation to postoperative patients with lung cancer still needs more clinical studies to explore, this study did not involve strength and endurance training, which is crucial in the concept of pulmonary rehabilitation.

## Data Availability

The data supporting this study can be obtained from the corresponding author [Jun Zhao]; As the research data involve patient privacy and informed consent, the data will not be disclosed.

## Abbreviations

NSCLC: Non-small cell lung cancer
VATS: Video-assisted thoracoscopic surgery
FEV_1_: Forced Expiratory Volume in One Second
FVC: Forced Vital Capacity
COPD: Chronic Obstructive Pulmonary Disease
6MWD: 6-Minute walk distance
